# Identification of epigenetic modifications that contribute to pathogenesis in therapy-related AML: Effective integration of genome-wide histone modification with transcriptional profiles

**DOI:** 10.1186/1755-8794-8-S2-S6

**Published:** 2015-05-29

**Authors:** Xinan (Holly) Yang, Bin Wang, John M Cunningham

**Affiliations:** 1 Section of Hematology/Oncology, Dept. of Pediatrics, Comer Children's Hospital University of Chicago, Chicago, USA

**Keywords:** computational biology, t-AML, EZH2, H3K27me3, SEMA3A

## Abstract

**Background:**

Therapy-related, secondary acute myeloid leukemia (t-AML) is an increasingly frequent complication of intensive chemotherapy. This malignancy is often characterized by abnormalities of chromosome 7, including large deletions or chromosomal loss. A variety of studies suggest that decreased expression of the *EZH2 *gene located at 7q36.1 is critical in disease pathogenesis. This histone methyltransferase has been implicated in transcriptional repression through modifying histone H3 on lysine 27 (H3k27). However, the critical target genes of EZH2 and their regulatory roles remain unclear.

**Method:**

To characterize the subset of EZH2 target genes that might contribute to t-AML pathogenesis, we developed a novel computational analysis to integrate tissue-specific histone modifications and genome-wide transcriptional regulation. Initial integrative analysis utilized a novel "seq2gene" strategy to explore largely the target genes of chromatin immuneprecipitation sequencing (ChIP-seq) enriched regions. By combining seq2gene with our Phenotype-Genotype-Network (PGNet) algorithm, we enriched genes with similar expression profiles and genomic or functional characteristics into "biomodules".

**Results:**

Initial studies identified *SEMA3A *(semaphoring 3A) as a novel oncogenic candidate that is regulated by EZH2-silencing, using data derived from both normal and leukemic cell lines as well as murine cells deficient in EZH2. A microsatellite marker at the *SEMA3A *promoter has been associated with chemosensitivity and radiosensitivity. Notably, our subsequent studies in primary t-AML demonstrate an expected up-regulation of *SEMA3A *that is EZH2-modulated. Furthermore, we have identified three biomodules that are co-expressed with *SEMA3A *and up-regulated in t-AML, one of which consists of previously characterized EZH2-repressed gene targets. The other two biomodules include MAPK8 and TATA box targets. Together, our studies suggest an important role for EZH2 targets in t-AML pathogenesis that warrants further study.

**Conclusion:**

These developed computational algorithms and systems biology strategies will enhance the knowledge discovery and hypothesis-driven analysis of multiple next generation sequencing data, for t-AML and other complex diseases.

## Introduction

The significance of non-coding DNA regulators in human disease has drawn increasing attention. For example, human non-coding regions contain collections of transcription factor binding sites and other regulatory elements called "cis-regulatory" regions. These cis-regulatory elements are sufficient to activate transcription in a defined spatial and temporal expression domain [1]. Cis-regulation can occur on either side of a target transcript and regulators can reside far from their regulatory targets [2]. However, identifying cis-regulatory elements and their domain-specific targets remains a major challenge for current computational biology. To address the challenge, we here perform a "sequence-regulator-network" study to integrate information from histone modification and transcriptional regulation. This method both generates and validates genomic hypotheses, and could have a broad impact in studying regulatory mechanisms of gene expression in systems biology. Here, we select therapy-related acute myeloid leukemia (t-AML) as a clinically significant context to apply the method.

T-AML, including therapy-related myelodysplastic syndrome, accounts for approximately 10 to 20 percent of myeloid malignancies [3]. T-AML complicates conventional chemoradiotherapies that are used to treat a variety of primary malignancies and is associated with a uniformly poor prognosis, with a median survival of six months [3]. Complete loss of chromosome 7 (-7) and 5 or partial deletion involving the long arm of chromosome 7 (del7q) are highly recurrent chromosomal aberrations in AML and t-AML.

Specific interest has focused on the link between common chromosome 7 abnormalities and the location of the EZH2 gene, the histone methyltransferase enhancer of Zeste homologue 2 present at position 7q36.1 [4]. Not surprisingly, *EZH2 *expression is significantly reduced in -7/del7q patients with myeloid disorders when compared with healthy controls [5]. What remains an enigma is the recently reported dual role of EZH2 in malignant cell development. EZH2 is a component of the polycomb group complex, which is vital for hematopoietic cell development. In normal cells, EZH2 suppresses its targets through depositing the histone modification mark H3K27me3 (trimethylation on lysine 27 of histone H3) [6]. In several epithelial cancers, overexpression of wild-type EZH2 has been found to promote tumor progression or metastasis [7,8]. However, inactive mutated *EZH2 *or its low expression in myeloid malignancy contributes to tumorigenesis by suppressing differentiation, thus directing cells toward a leukemic stem cell state [5,9,10]. Conflictingly, Xu et al. reported that *EZH2 *overexpression was associated with poor patient outcome in myeloid disorders and chemotherapy reduced expression of *EZH2 *[11].

These conflicting observations about EZH2 suggest a context-specific regulatory mechanism, which may be explained by plastic epigenetic modification [12]. Histone methylation is an important epigenetic modification in chromatin. The histone modification mark H3K27me3 reflects EZH2-involved Polycomb-mediated repression, and the deposition of H3K27me3 is development- stage-, and tissue-specific [13]. Thus, we studied the presence of H3K27me3 to understand the conflicting roles of EZH2 -- as either an oncogene or a tumor suppressor in different tumors. However, abnormal EZH2-associated regulation to particular target genes remains unclear in leukemia, specifically in t-AML.

Using ChIP-seq (Chromatin Immunoprecipitation Sequencing) of histone marks and other regulatory proteins, researchers can perform genome-wide searches for intergenic functional elements (including promoters and enhancers), but might also identify non-enhancers with similar signatures [14]. Therefore in this study, we evaluated whether the selective regions control the developmental expression of the target genes using transcriptomic measurements. To identify EZH2 target genes and their functional regions in t-AML, we developed a novel computational integrative analysis with histone modification of H3K27me3 and gene expression.

In the proposed integrative analysis, there are three distinguishing features: 1) a novel "seq2gene" strategy links genomic regions to more neighboring coding genes on both sides, 2) selective transcriptional and epigenetic data mining between cells, and 3) the prediction of "biomodules". The seq2gene strategy links genomic regions to a broad range of neighboring genes rather than the nearest one. The rationale is that enhancers can target long-range DNA targets, and often multiple enhancers (five or more) target the same genes [14]. The strategy then incorporates epigenetic regulatory patterns that differ between cell lines with disease-specific transcript alterations in t-AML. Finally, the selected target gene (seed) is associated with a group of genes, the "biomodule" that share similar expression patterns and genomic or functional characteristics, using our PGNet algorithm [15].

This integrative "sequence-regulator-network" study revealed *SEMA3A *(semaphoring 3A) as a novel target of EZH2-silencing in t-AML. The fact that *EZH2 *and *SEM3A3 *are inversely expressed *in vivo *is supported by previous data in mouse haematopoietic stem cells and human prostate cancer [16,17]. We predict that the loss of EZH2 silencing on *SEMA3A *augments sensitivity to both chemo- and radiotherapy, and thus may contribute to therapy-related AML pathogenesis. We also show that a group of *SEMA3A*-coexpressed genes, including *HOXA11*, are up-regulated in t-AML and have been reported as EZH2 targets. We expect further validation both *in vitro *and *in vivo*.

## Result

### Identification of SEMA3A, a loss-of-EZH2-mediated silencing gene in leukemia

To systematically screen functional elements of histone modified EZH2 targets in leukemia, we used the data in the Encyclopedia of DNA Elements (ENCODE, http://genome.ucsc.edu/ENCODE/) Project [18]. We identified 104,370 genomic regions that are enriched for both EZH2 and H3K27me3 in the leukemia cell line (K562) and 53,360 regions in the lymphoblastoid cell line (GM12878). The large number of enriched regions (peaks) suggests substantial downstream effects of EZH2 repression. EZH2 occupancy and presence of H3K27me3 at promoters directly silences the transcription of targeted genes, which has been observed in leukemia and other tumors [19,20]. Given this, we predict that genes adjacent to these non-coding regions are EZH2 repressive target candidates.

**Figure 1:**
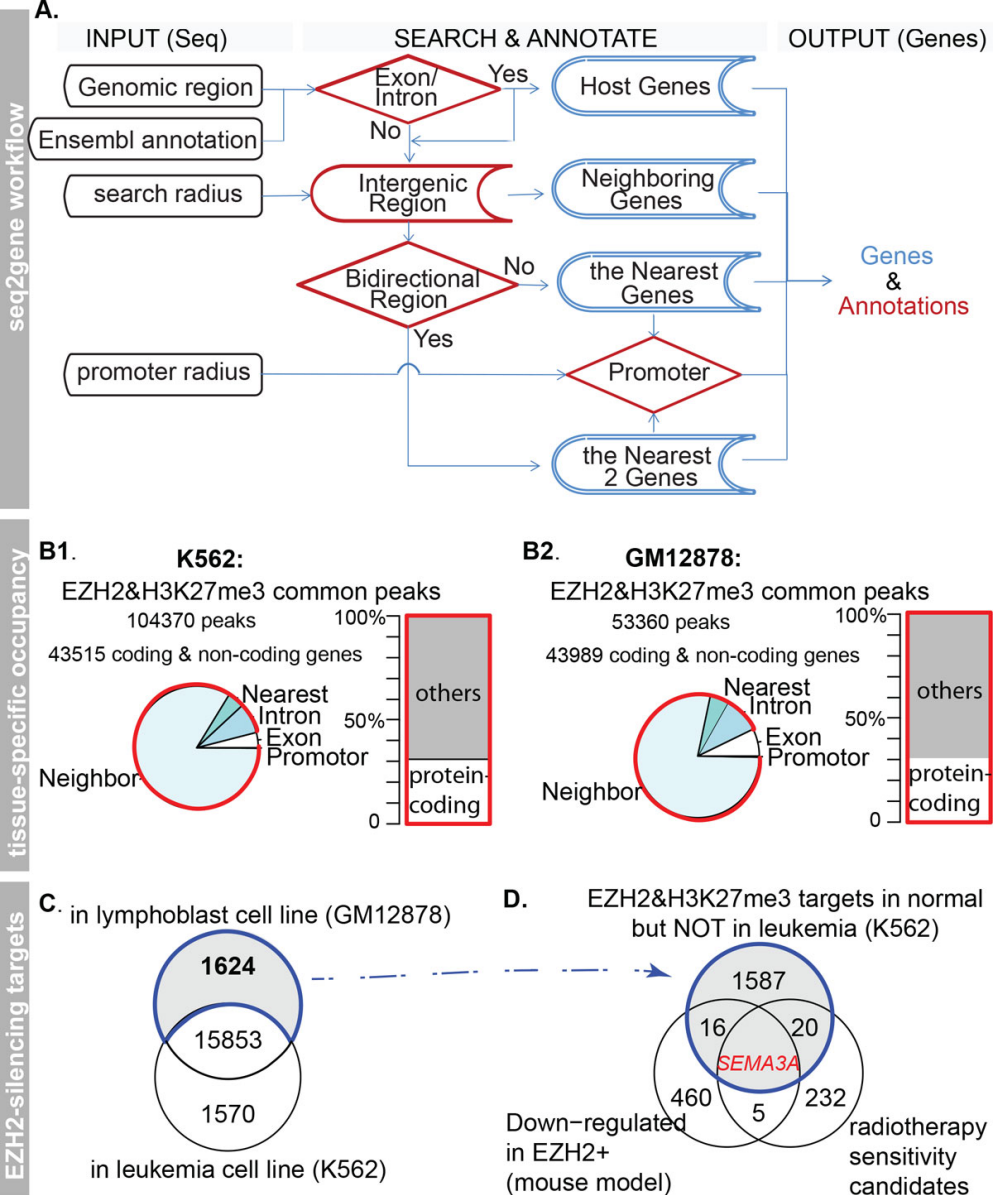
**Identification of SEMA3A, a loss-of-EZH2-mediated silencing gene in leukemia**. A) The seq2gene strategy links genomic regions to coding genes with annotation. B) For both cell lines, more than 90% of the EZH2 and H3K27me3 co-binding regions are linked to intergenic coding and non-coding genes (red arc and box), 31% of which are coding-genes. C) Leukemic and lymphoblastic cell-specific EZH2 and H3K27me target genes. D) SEMA3A loses EZH2-silencing in leukemia but with EZH2-dependent expression changes in two independent experiments (the white circles). The gray circle represents 1624 predicted co-binding sites specific to lymphoblastoids identified in panel C.

To discover target candidates, we associated coding genes residing in a given search radius to the identified regions, using a "seq2gene" mapping strategy (Figure 1A). Seq2gene considers the possibility that genes in both directions from each intergenic cis-regulatory element may fall under control, given the observation that enhancers reside on average 120-thousand base pairs (bps) away from their regulatory targets and act independently of their orientation in mammals [2,14,21]. This consideration resulted in mapping around 90% of EZH2 and H3K27me3 co-mediated loci to neighboring genes within an arbitrary distance of 150k-bp on both sides, of which only 31% are coding genes (Figure 1B, the ENSEMBL Hg19 assembly and definition).

To focus on EZH2-mediated coding-gene silencing that is specific to leukemia, we compared the candidates in leukemia with lymphoblastoids. Only around 10% of the identified ~17,400 EZH2 repressed coding-gene candidates are disease-specific, resulting in 1624 genes specific to lymphoblastoid but not leukemia (Figure 1C). Gene Ontology enrichment analysis suggests a loss of leukemia-specific repressive control on the molecular function termed "hematopoietin/interferon-class (D200 domain) cytokine receptor binding" (GO:0005126, FDR = 0.0017, count = 12), reflecting a cell quiescence-involved, generic cancer metastatic mechanism [22,23].

We subsequently identified *SEMA3A *as an EZH2 repressive target of interest. This identification is derived from sequence-based analysis and transcriptional evidence (Figure 1D). From the ChIP-seq peaks in the lymphoblastoid cell line, we observed EZH2 occupancy and presence of H3K27me3 adjacent to the transcription start site of *SEMA3A *and 20 other genes (Additional file: Table S1). These 21 genes significantly over-represent genes highly expressed in prostate cancer cells after knockdown of EZH2 [16] (p = 0.009, OR = 5.8) and genes down-regulated in fibroblasts expressing mutant forms of ERCC3 after UV irradiation [24] (P = 0.0046, OR = 7.1). As ERCC3 could help increase the sensitivity of cancer to radiation therapy, loss of EZH2-repression of these ERCC3 targets in t-AML indicates an increased radiosensitivity. Specifically, the transcriptional expression of *SEMA3A *is negatively *EZH2*-dependent in both human cancer and mouse model *in vivo*. For example, Merchan et al. generated mouse models that allow gain-of-function of Ezh2 in the haematopoietic system [17], and we identified a 2.2-fold decrease of *Sema3a *expression in Ezh2*+ *mice compared with wild-types (Q-value = 0.05, the limma test [25]). However, in the leukemia cell line, a peak with both EZH2 and H3K27me3 enrichment has not been observed in the human genomic region within 150kbp distance to *SEMA3A*, suggesting a leukemia-specific loss of EZH2-silencing on *SEMA3A*.

High-expression of SEMA3A may contribute to t-AML pathogenesis by augmenting chemosensitivity and radiosensitivity

We observed the loss of EZH2 and H3K27me3 enrichment on the *SEMA3A *promoter in leukemia (Figure 1C-D) and hypothesized that this loss rescues *SEMA3A *expression and facilitates leukemogenesis after chemo- or radiotherapy.

To validate this hypothesis, we investigated CD34+ cells from 28 t-AML patients including 8 with -7/del7q abnormality, and 24 normal controls (Table 1). Expression profiles of samples collected from different laboratories were adjusted for batch effects [26] (Additional file: Fig. S1) and then t-AML samples were compared to normal controls. There are 370 significantly up-regulated and 686 down-regulated genes (Q-value<0.05, FC≥2 or ≤0.5) when comparing -7/del7q t-AML samples with controls.

**Table 1:** Six studies pertaining to CD34+ cells in t-AML and normal controls.

	GSE24006	GSE30377	GSE17054	E-TABM-978	Qian	GSE23025	sum
	
Journal	leukemia	Nat. Med	PNAS	Cancer Cell	PNAS	Cancer Cell	
	
year	2011	2011	2009	2011	2002	2011	
	
Platform	Hgu133+2	Hgu133a	Hgu133+2	HsHT-12	Hgu95av2	Hgu133+2	
	
PMID	21177505	21873988	19218430	21251617	12417757	22094254	
t-MDS/tAML CD34+ progenitor (BM), -5/del5q					4	1	**28**
	
t-MDS/tAML CD34+ progenitor (BM), -7/del7q					3	3	
	
t-MDS/tAML CD34+ progenitor (BM), -5/del5q or -7/del7q					2		
	
t-MDS/tAML CD34+ progenitor (BM), normal 5 and normal 7					7	8	

normal progenitor (CD34+, BM)					2*		**24**
	
normal HSC+ (CD34+CD133+, BM)							
	
normal HSC+ (Lin-CD34+CD38-, PB)		3					
	
normal HSC+ (Lin-CD34+CD38loCD36-, PB)		3					
	
normal HSC+ (Lin-CD34+CD38-CD90+, PB or BM)			4				
	
normal HSC+ (Lin-CD34+CD38-CD90+CD45RA-, PB)	3						
	
normal HSC+ (Lin-D34+CD38-CD90+CD45RA-, BM)	4			5			

* Among 3 author collected samples, the one from a patient with breast cancer was excluded and the other two were included.

PBSC: peripheral blood stem cells; BM: bone marrow samples

**Figure 2:**
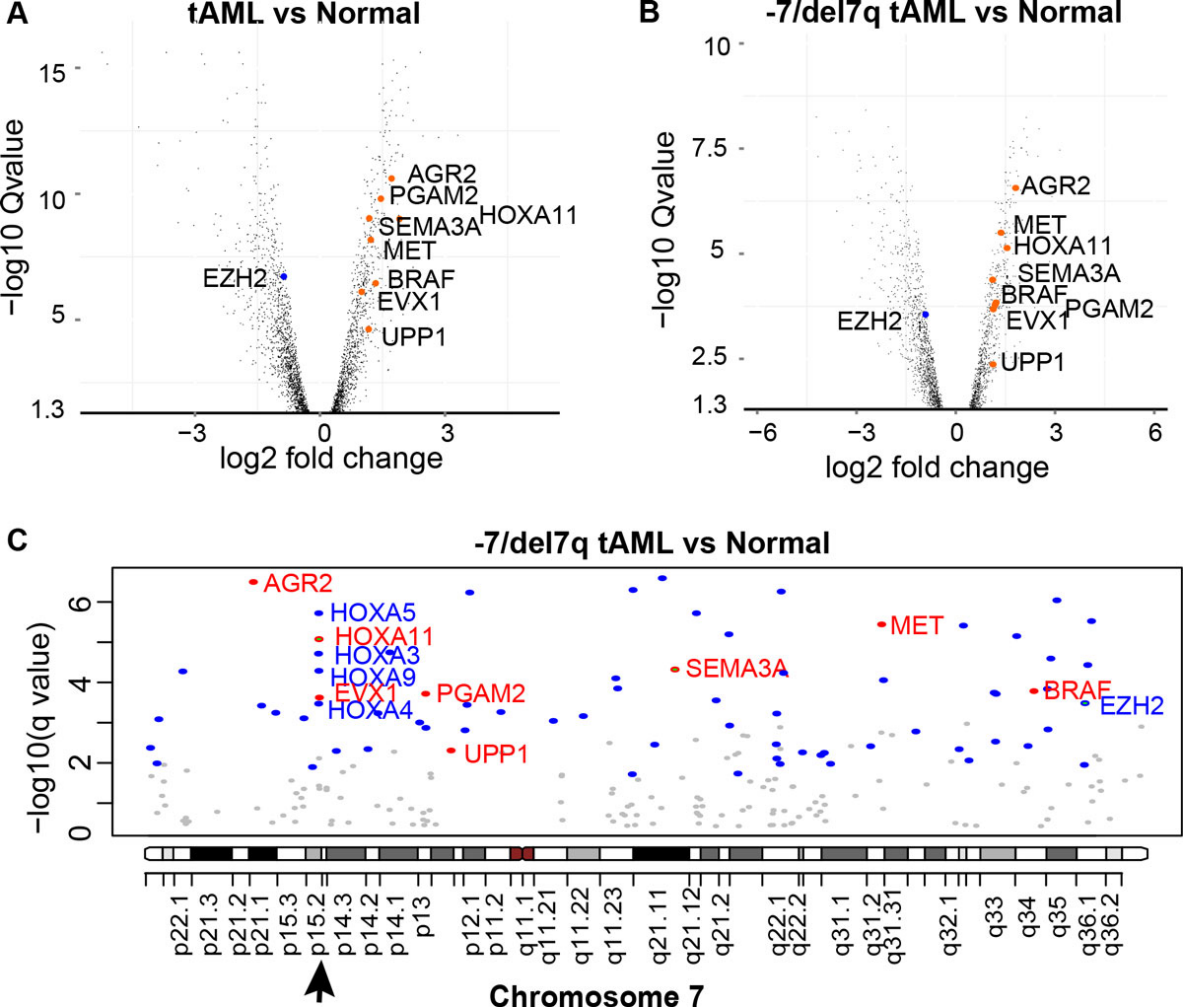
**Gene differential expression in t-AML compared with controls**. A) Significantly differentially expressed genes in t-AML when compared with normal HSC+ cells (Q-value<0.05). B) Significantly differentially expressed genes in -7/del7q t-AML when compared with normal HSC+ cells (Q-value<0.05). C) The significantly up-regulated (red) or down-regulated (blue) genes in −7/del7q t-AML, residing on chromosome 7. The non-significant genes are in gray. The HOX family genes in 7p15.2 consist of both up- and down-regulated genes.

Significantly, *SEMA3A *shows up-regulation in patients with t-AML (Q-value = 1.1e-9, FC = 2.3, Figure 2A), even in patients with deletion of chromosome 7 or loss of chromosome 7q (FC = 2.2, Q-value = 4.6e-5, Figure 2B). In contrast, *EZH2 *was significantly down-regulated in t-AML (Q-value = 2.3e-07 and 0.00032, FC = 0.55 and 0.54, respectively). Besides *SEMA3A*, there are another 7 genes (*AGR2, EVX, HOXA11, MET, PGAM2, BRAF*, and *UPP1*) residing on chromosome 7 are significantly up-regulated in t-AML even with -7/del7q abnormality (Figure 2B-C). Three of them (*HOXA11, MET, BRAF*) are potential oncogenes currently being observed for common copy-number gains in a meta-analysis of copy number alterations across a panel of different cancer cell lines and tumor samples [27]. Their high expression suggests that a loss of EZH2 regulation dominates the expression changes of *SEMA3A *and *HOXA11*.

**Figure 3:**
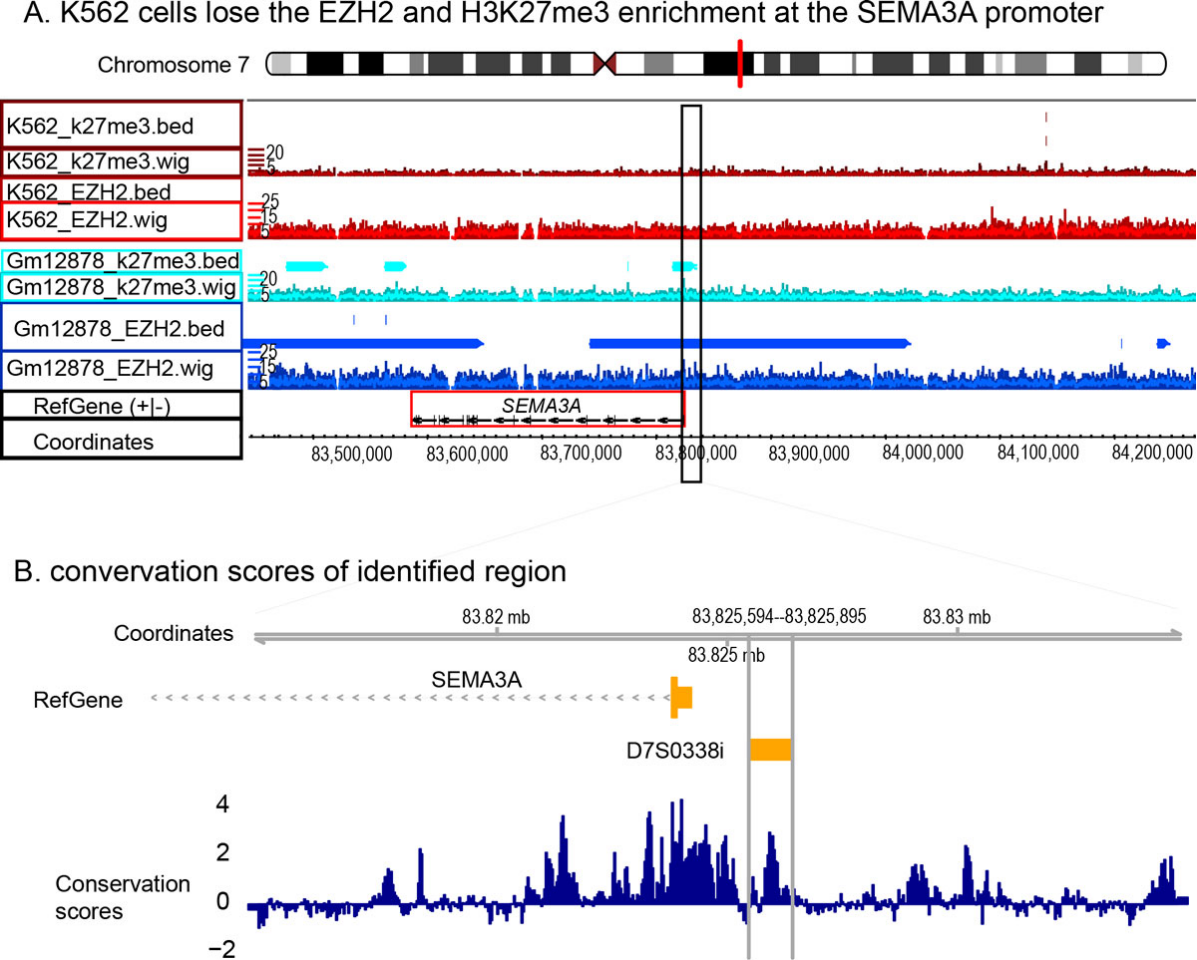
**Genomic view of the *SEMA3A *promoter**. A) The SEMA3A promoter is enriched with EZH2 and H3K37me3 in the lymphoblastoid cell line (GM12878) but not in the leukemic cell line (K562). B) This promoter region loses EZH2-silencingand covers a microsatellite marker, D7S0338i, which is significantly associated with acute adverse effects during radiotherapy in cancer patients.

More importantly, significantly higher expressed *SEMA3A *was previously reported in chemosensitive cancers than in chemoresistant tumors [28,29]. Additionally, an identified EZH2-H3K27me3-enriched promoter region of *SEMA3A *(Chr7:83,814,596-83,835,002) covers a microsatellite marker that is significantly associated with acute adverse effects following radiotherapy in cancer patients [30] (Figure 3A). Note that the Phylop score, corresponding to cross-species genome conservation [31], is relatively high within this marker (Figure 3B), suggesting that it is functionally important. This observation agrees with the previous finding that biochemical, evolutionary, and genetic approaches provide complementary information for defining functional DNA segments [32]. This intergenic region (D7S0338i, chr7:83,825,594-83,825,895, Hg19 assembly) is 1.5k-bp upstream of the transcription start site of the *SEMA3A *gene. Evidence from normal skin cells has proven that *SEMA3A *knockdown enhances radiation resistance, suggesting an increased radiosensitivity with loss-of-silencing on *SEMA3A *in leukemia [30].

The stem cell self-renewal HOX gene family has been described as a major downstream target of EZH2. Unlike *HOXA11*, the other four HOX family genes (*HOXA3, HOXA4, HOXA5*, and *HOXA9*) are down-regulated when comparing -7/del7q t-AML cells against controls (Q-value<0.001, fold change (FC)<0.4, Figure 2C). Of note, sublethally irradiated Hoxa9-/- mice exhibited prolonged suppression of hematopoiesis and developed persistent pancytopenia [33], indicating an enhanced sensitivity to ionizing irradiation in t-AML cells with deficient *HOXA9*.

### Genes co-regulated with SEMA3A in t-AML are enriched in EZH2 repressed targets

**Figure 4:**
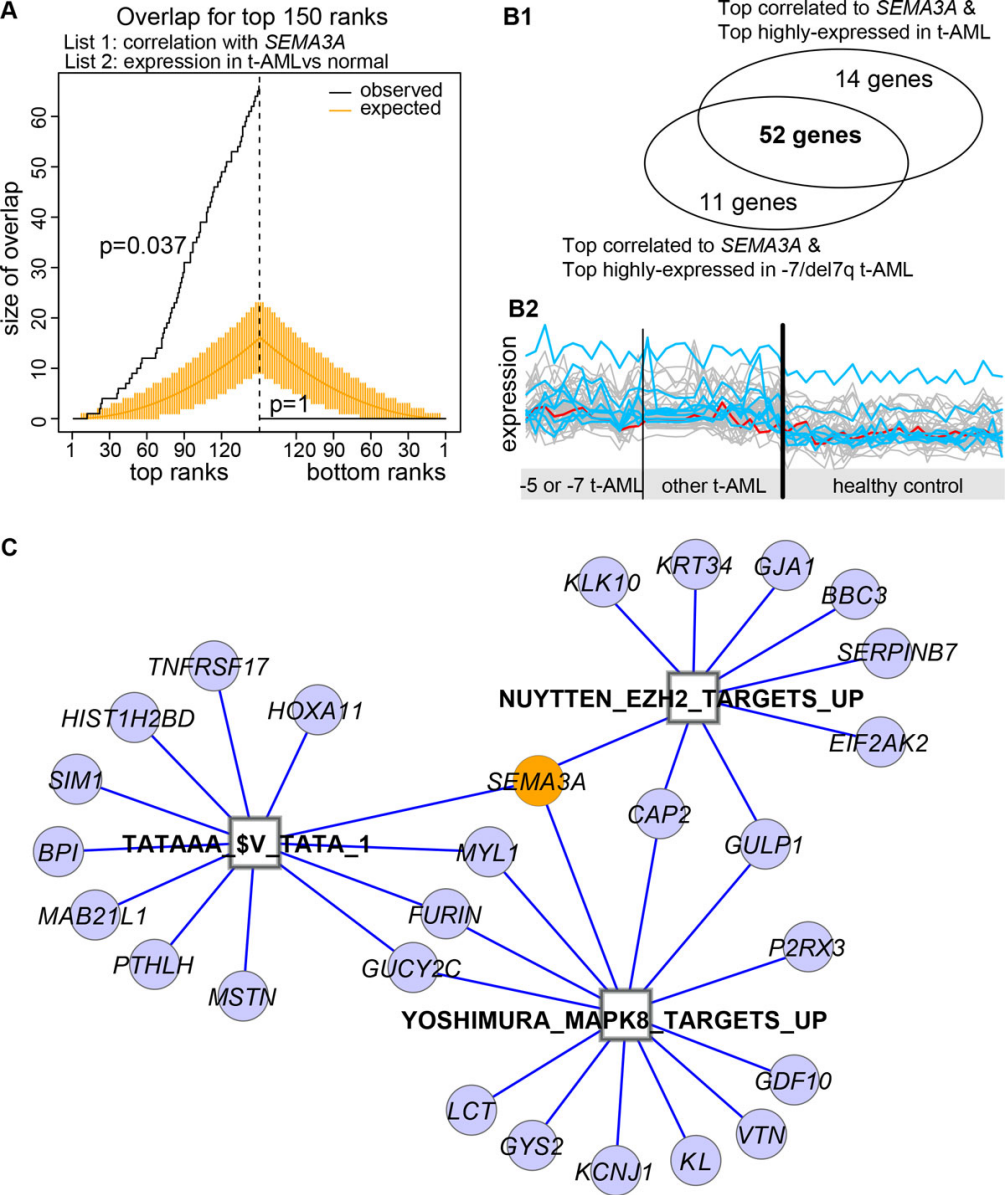
**PGNet identifies three biomodules**. A) The significance exists among top ranks but not the bottom ranks when comparing orders of two genome-wide gene expression statistics, one is the correlation with SEMA3A and the other is the differential expression between t-AML and control. The black line records the overlap among the top 150 ranks, while the orange line is the estimated overlap by chance. In addition, the expected overlap and 95% confidence intervals derived from a hypergeometric distribution are shown. B) 52 intersecting genes were commonly identified when the second statistic of the PGNet algorithm comparing t-AML to controls or -7/del7q t-AML to controls (subpanel 1). The expression values of these 52 genes (gray) are lines along with t-AML and control samples in subpanel 2, the red line sketches SEMA3A and the blue lines outline the other EZH2 targets in prostate cancer. C) Functional enrichment analysis identifies three sub-sets of genes from SEMA3A and the 52 genes, each sub-set significantly over-represented one MSigDB defined functional gene-sets (p < 0.001, count>5). Genes are marked as circles, and their functional terms are marked as squares. The expression pattern of EZH2 targets is highlighted as blue lines in Fig B2.

To further study *SEMA3A *function in t-AML, we investigated the functional enrichment among genes sharing an expression pattern with *SEMA3A*. Previously, we developed a phenotype-genotype network analysis (PGNet) algorithm to define a group of genes that share significant concurrence of expression pattern with respect to sample grouping (a phenotype of interest) and gene regulation (a genotype of interest) [15]. The PGNet algorithm was successfully applied to identify epigenetic regulators, despite the fact that transcriptional signatures of epigenetic regulation is subtle [34,35], thus vetting the method for our similar such application. Using PGNet, we defined 66 genes that meet two criteria among 28 patients and 24 controls: *1*) systematic co-expression with *SEMA3A*, and *2*) higher expression in t-AML than in normal samples (among the top 150 ranks for both statistics). The similarity of the orders of these two gene expression statistics are significant at the top ranks but not the bottom ranks (empirical p = 0.037, permutation times = 1000). Specifically displayed in Figure 4A are the numbers of genes in which these two top ranks overlap. The overlap size is drawn as a step function over the respective ranks. Top ranks correspond to up-regulation in t-AML and positive correlation with *SEMA3A*, and bottom ranks correspond to down-regulation and negative correlation. The PGNet algorithm also identified 63 genes when comparing -7/del7q t-AML patients (n = 8) to controls and modifying the second criteria appropriately (top 150 for both statistics, empirical p = 0.024). Of note, 52 genes overlap between the two defined gene-sets (Figure 4B, Additional file: Table S2), suggesting a common transcriptional regulatory mechanism in t-AML with or without chromosome 7 loss.

Importantly, we found three functional biomodules among the 52 genes correlated with *SEMA3A *(Fisher's exact test, p ≤ 0.001, count>5, Figure 4C, Table 2). One module includes nine genes (*EIF2AK2, GULP1, CAP2, GJA1, SEMA3A, KRT34, SERPINB7, BBC3, KLK10*, p = 0.001) that were up-regulated in PC3 cells (prostate cancer) after knockdown of EZH2 by RNAi [16]. Shown in Figure 4B2, the expression patterns of these EZH2-repressed genes (blue lines) are correlated with *SEMA3A *(red line) and are higher in t-AML than in controls. These genes constitute a biomodule representing the expression pattern of a loss of EZH2-silencing. Another identified module is 12 TATA box binding protein genes with promoter regions (defined as ±2kb around the transcription start site) containing the motif TATAAA, including *SEMA3A *and *HOX11A *(p = 4.6e-4, Figure 4C). The HOX gene family is recognized as a major downstream target of EZH2 [12]. This supports the notion of *SEMA3A*-correlated *HOX11A *up-regulation in t-AML. The third and most significant biomodule is 13 genes up-regulated in vascular smooth muscle cells by MAPK8 (p = 1.6e-5) [36]. *MAPK8*, also known as JNK1, encodes many transcripts and is activated by various cell stimuli. The biomodule of MAPK8-induced and t-AML highly expressed genes supports the observation that *MAPK8 *is involved in carcinogenesis [37]. The regulatory mechanism linking *MAPK8 *and *SEMA3A *remains unclear. Additionally, a previous study shows that the EZH2 "loss-of-function" mutation contributes to formation of the leukemic stem cell by mediating self-renewal of myeloid progenitors [9], indicating that the loss of EZH2 silencing on *SEMA3A *contributes to leukemogenesis in t-AML.

**Table 2:** Three functional and transcriptional biomodules.

MsigDB definition					Fisher's exact test		
**Gene-set**	**Category**	**Description**	**Pubmed**	**Size**	**P**	**OR**	**#**

YOSHIMURA_MAPK8_TARGETS_UP	CGP	Genes up-regulated in vascular smooth muscle cells (VSMC) by MAPK8 (JNK1)	16311603	1305	1.6e-5	5.0	13

TATAAA_V$TATA_01	Motif	Genes with promoter regions [-2kb,2kb] around transcription start site containing the motif TATAAA which matches annotation for TAF TATA	NA	1296	4.6e-4	3.7	12

NUYTTEN_EZH2_TARGETS_UP	CGP	Genes up-regulated in PC3 cells (prostate cancer) after knockdown of EZH2 by RNAi.	17724462	1037	1.1e-3	4.0	9

CGP: Gene-sets represent expression signatures of genetic and chemical perturbation; OR: odds ratio; #: count.

## Method

### Data

The chromatin immunoprecipitation sequencing (ChIP-seq) peaks for EZH2 occupancy and presence of H3K27me3 were downloaded from the Encyclopedia of DNA Elements (ENCODE, Release 3, hg19 assembly) [38]. We focused on human blood cell lines: K562 from leukemia and GM12878 from lymphoblastoid cell of a female donor.

We collected published expression levels of CD34+ progenitor cells from 28 t-AML patients and 24 healthy control samples (Table 1) [39-43]. Samples from patients with breast cancer or lymphoma before the development of t-AML were excluded from the control group [39,40].

Functional gene-sets were defined by the MsigDB database [44].

### Identifying target genes of transcription regulators or histone marks

**Figure 5:**
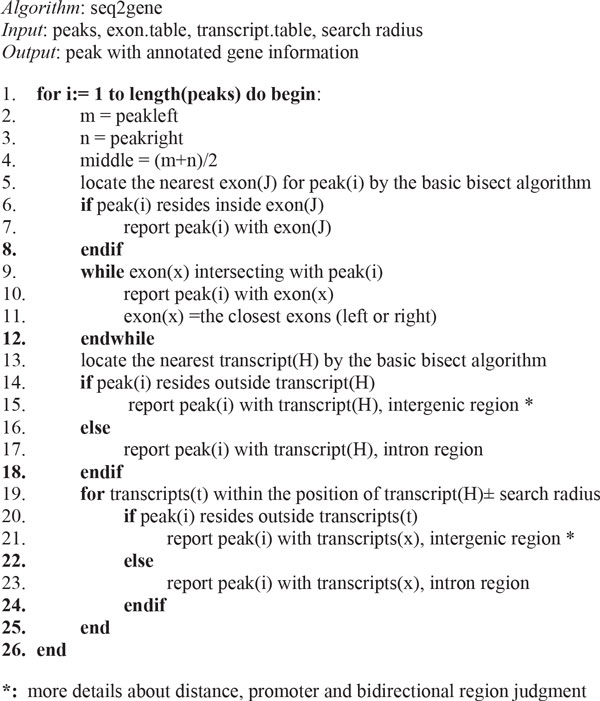
**Pseudo code of the seq2gene algorithm**.

To define the EZH2 repressed targets in a cell line, we performed a two-step analysis: 1) intersecting significant ChIP-seq peaks of EZH2 and H3K27me3 to find the common enriched genomic regions; 2) finding candidate target genes and annotating them using the seq2gene strategy (Figure 5). Note that the bisection method is used to perform a binary search among exon and transcript annotations. To perform a search with respect to exon and transcript separately, we have prepared the "exon.table" and "transcript.table" files based on the ENSEMBL general feature format for end users (Figure 5). Both files use ENSEMBL IDs as the key index. This analysis was performed for each cell line separately.

### Analyzing transcriptomic data

*Data pre-processing*. The normalized expression profiles were downloaded from the Gene Expression Omnibus (GEO, http://www.ncbi.nlm.nih.gov/geo/) or ArrayExpress (http://www.ebi.ac.uk/arrayexpress/). The raw data from the Qian's dataset was processed using the global rank-invariant normalization (GRSN) [45]. Gene expression profiles were log2-transformed if authors hadn't already done so. When collapsing probes to genes for each dataset, probes with the same Entrez IDs were collapsed to the maximum mean expression per gene using an empirically recommended method [46] and the Bioconductor annotation package biomaRt (v2.18.0), resulting in a set of 8442 human Entrez genes measured across all cohorts. The batch effects due to multi-datasets were removed by an empirical Bayes method using the Bioconductor package sva [26,47] (Additional file: Fig. S1). Then a smaller data space of 4221 genes was considered for the following analysis by keeping the half of genes with the highest interquartile range [48].

### Identifying biomodule

*Step 1 to build biomodules: transcriptional association*. To identify the potential upstream regulators or down-stream targets of the genes that drive the tumorigenesis in t-AML, we applied the PGNet method [49]. PGNet evaluates the similarity of the gene orders between two independently ordered lists. Specifically, we used PGNet to compare genome-wide correlated expressions with a seed gene and the differential expressions between two sample groups and yielded a regulatory network of genes that are mutually associated. To identify the potential downstream regulatory targets of *SEMA3A*, we inputted it as a seed gene together with the phenotype information (t-AML: n = 28, normal control: n = 24) into the PGNet system to infer a regulatory network [49]. Differential expression (DE) was estimated using the Bioconductor package Limma [25] and co-expression (CE) was evaluated by the Pearson correlation test on the log-transformed data. The resulting p-values were adjusted by a Q-value for false discovery rate in the multiple testing problem [50,51]. The significance of the similarity between the two statistics (coefficient and fold change) was estimated using an empirical p-value based on permutation (n = 1000), using the Bioconductor package OrderedList [35]. We also tested the differential expression between -q/del(7q) t-AML (n = 8) and normal controls to determine whether a regulatory biomodule for t-AML is consistent despite loss of chromosome 7.

*Step 2 to build biomodules: genomic or functional enrichment*. The over-representation of functional gene-sets among the identified biomodule was evaluated using a conditional hypergeometric distribution test (Additional file: Fig. S2). Note that only the true background, the common genes within MSigDB and those covered by the experiment, were used for the test. We used a threshold of Q-value<0.001 and count>5 for significance over MSigDB defined functional genesets [44].

## Discussion

The ENCODE project increasingly produces genomic data on transcription factor binding, chromatin structure and histone modification. Interpreting transcriptional regulation in relation to chromatin modifications has been recognized as a powerful strategy to discover and understand intergenic regulatory elements (reviewed by Kellis et al. [32]). However, ChIP-seq enrichment for chromatin modification and differential expression for transcriptional control may provide complementary information. Thus, the simple overlap strategy, that we here used, may discover only limited targets [52]. We expect additional strategies to reveal more candidates than just a single locus to elucidate genomic function in human biology and diseases. To address this challenge, we piloted the application of the PGNet algorithm to build genomic and functional related as well transcriptionally associated gene-sets (biomodules). The identified biomodule of EZH2-suppressed targets that up-regulated in t-AML in conjunction with *SEMA3A*, in turn, evaluated our hypothesis that *SEMA3A *is a critical EZH2-silencing target.

The reduced *EZH2 *expression in t-AML parallels reduced expression observed in primary AML or pre-leukemia, which was previously found in 78% of patients carrying either *EZH2 *inactive mutation or -7/del7q involving the *EZH2 *locus [9]. Importantly, primary AML patients who have lower *EZH2 *expression (either spliceosomal mutants or -7/del7q) show decreased H3K27 trimethylation and increased chromatin relaxation at specific gene loci accompanied by higher transcriptional activity [9]. Using the proposed "sequence-regulator-network" strategy, we identified *SEMA3A *as a new such gene target that loses epigenetically modified *EZH2 *silencing in t-AML. The identified gene locus covers a radiation sensitivity mark revealed by genome-wide association study [53]. However, *HOXA9 *was found to be overexpressed in cases of either *EZH2 *mutations or -7/del7q when compared to *EZH2 *wild-type [9], which differs from the observation in our t-AML samples. This observation indicates a commonly reduced histone modification and alternative leukemogenic regulation on *HOXA9 *between t-AML and primary AML. Both involve EZH2 and potentially some of its DNA-binding cofactors. We expect further validation both *in vitro *and *in vivo*.

A literature review suggests two possible mechanisms to explain why this epigenetically modified up-regulation of *SEMA3A *contributes to pathogenesis in therapy-related AML. The first could be a reduction of DNA repair capacity, given that *Sema3A *suppresses angiogenesis and migration in mice models [54,55] and thus triggers the sensitivity of leukemic cells to apoptosis signal [56], possibly via a *MAPK8 *regulated pathway (Figure 4C). On the other hand, a previous study found that *Sema3A *counteracted chemotherapy-induced activation of epithelial-mesenchymal transition (EMT) by improving cancer tissue oxygenation and extending the vascular normalization [57]. Therefore, the second mechanism could be the production of cancer stem cells (CSCs), given that cells that undergo EMT gain stem cell-like properties [58].

In summary, we predicted EZH2-silencing targets and their functions in t-AML by performing a novel computational integrative analysis. The analysis incorporates chromatin-based epigenetic regulation patterns in different cell lines with transcriptional expression alteration pertaining to t-AML. This integrative analysis promises to reveal novel functional elements in a complex and versatile regulatory system behind target gene selection and their tissue-specific expression.

## Competing interests

The authors declare that they have no competing interests.

## Competing interests

The authors declare that they have no competing interests.

## Supplementary Material

Additional file 1 **Table S1**. The 21 candidate genes predicted in Figure 1D. They exhibit not only microsatellite markers associated with radiosensitivity but also genomic regions enriched with EZH2 and H3K37me3 in the lymphoblastoid (GM12878) only, not the leukemic cell line (K562). Genomic loci are based on the hg19/GRCh37 assembly. **Table S2**. 52 SEMA3A dependently differentially expressed genes in t-AML. **Table S3**. ENCODE data resource used in this study. **Figure S1**. Correction of batch effects. A) There are batch effects when integrating samples from different datasets, showing by the first two principal components derived from all genes. B) The dataset-dependent batch effects are removed after the correction. In both panels, one dot is one sample colored by the datasets. **Figure S2**. Conditional hypergeometric distribution test. Note that the test uses the common genes (A+B+C+D) covered by both MSigDB and an experiment of interest. fGS: functional gene-set; DE: differentially expressed. Click here for file

## Declarations

Publication of this article has been funded by the National Institutes of Health [R21 CA167305].

This article has been published as part of *BMC Medical Genomics *Volume 8 Supplement 2, 2015: Selected articles from the 4th Translational Bioinformatics Conference and the 8th International Conference on Systems Biology (TBC/ISB 2014). The full contents of the supplement are available online at http://www.biomedcentral.com/bmcmedgenomics/supplements/8/S2.
