# Supplementary material for: Identification of epigenetic modifications that contribute to pathogenesis in therapy-related AML: Effective integration of genome-wide histone modification with transcriptional profiles
Source: BMC Med Genomics. 2015 May 29;8(Suppl 2):S6. doi: 10.1186/1755-8794-8-S2-S6 (PMC4460748; doi:10.1186/1755-8794-8-S2-S6)
Supplement: Additional file 1 — Table S1. The 21 candidate genes predicted in Figure 1D. They exhibit not only microsatellite markers associated with radiosensitivity but also genomic regions enriched with EZH2 and H3K37me3 in the lymphoblastoid (GM12878) only, not the leukemic cell line (K562). Genomic loci are based on the hg19/GRCh37 assembly. Table S2. 52 SEMA3A dependently differentially expressed genes in t-AML. Table S3. ENCODE data resource used in this study. Figure S1. Correction of batch effects. A) There are batch effects when integrating samples from different datasets, showing by the first two principal components derived from all genes. B) The dataset-dependent batch effects are removed after the correction. In both panels, one dot is one sample colored by the datasets. Figure S2. Conditional hypergeometric distribution test. Note that the test uses the common genes (A+B+C+D) covered by both MSigDB and an experiment of interest. fGS: functional gene-set; DE: differentially expressed. [file 1755-8794-8-S2-S6-S1.pdf]

Table S1. The 21 candidate genes predicted in Figure 1D. They exhibit not only microsatellite markers associated with radiosensitivity but also genomic regions enriched with EZH2 and H3K37me3 in the lymphoblastoid (GM12878) only, not the leukemic cell line (K562). Genomic loci are based on the hg19/GRCh37 assembly.

**Symbol**	**Entrez ID**	**Chr**	**Coordinate#**	**EZH2_H3K27me3_common_peak_GM12878**	**Microsatellite**	**Marker***
AL359693.1	NA	chr6	82262307-82262537	82459829-82459975; 82459875-82460075; 82459452-82461027; 82460595-82460769	81982490-81982915	D6S0876i
ALCAM	214	chr3	105085557-105295757	105071969-105072970	102991462-102991819; 103914598-103915045	D3S0313i; D3S0923i
ARAP2	116984	chr4	36067620-36245979	34687579-34688660; 35855103-35856191; 35855841-35856029; 35913637-35914220; 35914671-35915174; 35925186-35947016; 35977237-35977445	30825655-30825824	D4S0132i
DTWD2	285605	chr5	118172569-118324240	117585281-117585485; 117894195-117894625; 117984519-117990302; 118064203-118064707; 118074880-118075007; 118115702-118126236; 118154129-118159824; 118004131-118031546; 118080151-118098483	117007981-117008433	D5S0292i
GAS7	8522	chr17	9813926-10101868	10038638-10039762; 10048312-10050134; 10050670-10050890; 9993669-9994796; 10003540-10013309	9823237-9823434	D17S945
MAD2L1	4085	chr4	120980579-120988013	120923526-120931558; 120962492-120963879; 121006202-121020400; 121079002-121079507; 121079189-121080400; 121079869-121080567	121837206-121837627	D4S0813i
NALCN	259232	chr13	101706130-102068813	101709088-101709257; 101734212-101736636; 101738981-101745330; 101746250-101755519; 101765748-101790711; 101799627-101809665; 101844403-101844548; 101910175-101911279; 101917457-101927502; 101981195-101981317; 102049494-102050047; 102069394-102069733; 102069260-102069968; 102109803-102127094; 102147643-102163779; 102165256-102175242; 102185238-102190168; 102212659-102220495; 102258870-102262671; 102268121-102276241; 102280262-102284601; 102295726-102302465	101340247-101340636	D13S0138i
PCDH7	5099	chr4	30722037-31148423	30724966-30726022; 30717649-30724028	30825655-30825824	D4S0132i
PRDM5	11107	chr4	121613068-121844021	121629180-121654804; 121680761-121714056; 121762723-121763985; 121781859-121781989; 121762881-121782690; 121785978-121786213; 121832280-121854710; 121896803-121899433; 121962766-121962873; 121962617-121964378; 121976324-122007962	121837206-121837627	D4S0813i
PRDM9	56979	chr5	23509577-23527777	22852777-22854850	23471354-23471799	D5S0803i
PTPRD	5789	chr9	8314246-10612723	7797975-7821296; 8845557-8879669; 8334557-8740553; 9634119-9639583; 9798899-9810311; 9580950-9588272; 9895078-9909248; 10610941-10615959	8745688-8746162	D9S0242i
RP11-67H2.1	NA	chr8	114627733-114629187	114443095-114451689	116236473-116236952	D8S0335i

SEMA3A	10371	chr7	83587659-83824217	83266888-83300743; 83563276-83583313; 83564151-83564357; 83564251-83564364; 83775344-83775556; 83814596-83835002	83825596-83825895	D7S0338i
SEMA3D	223117	chr7	84624872-84751247	83814596-83835002; 84642876-84650279; 84656826-84663660; 84811783-84818143; 86267959-86282696; 86286313-86301860; 86314131-86314484; 86327951-86336521	83825596-83825895	D7S0338i
SLC9A8	23315	chr20	48429250-48508779	48413535-48414229; 48415123-48415269; 48404691-48418876; 48417323-48417533	48281896-48282042	D20S0325i
TECRL	253017	chr4	65144177-65275178	66532846-66535055	65285295-65285463	D4S0317i
TLE1	7088	chr9	84198598-84303596	84301279-84303078; 84305943-84306479	83697608-83697713	D9S0704i
TLE4	7091	chr9	82186688-82341796	81586577-81592581; 81597878-81609641; 81615960-81616248; 81617689-81628169; 81636297-81647290; 81650494-81650764; 81766571-81789425; 81870865-81871931; 81876283-81877285; 82014747-82015757; 82074394-82074907; 82084629-82086052; 82091665-82101791; 82140626-82141922; 82163828-82183188; 82411564-82411695; 82407212-82447508; 82462662-82463164; 82462997-82464021; 82480284-82495782; 82496810-82498253; 82515323-82524904	83697608-83697713	D9S0704i
TMEM261	90871	chr9	7796491-7799799	7407569-7407817; 7409861-7409968; 7448351-7479287; 7532220-7532425; 7491401-7550726; 7551672-7576703; 7589444-7589993; 7599515-7609593; 7658749-7658857; 7676998-7677206; 7677098-7677208; 7724830-7731362; 7733573-7734583; 7761194-7761530; 7745561-7756360; 7768213-7782167; 7806531-7806636; 7806533-7806736; 7787493-7787987; 7797975-7821296; 7952948-7957479; 7968444-7973420; 7973416-7973420	7117471-7117725	D9S0416i
TYRP1	7306	chr9	12693386-12710266	8845557-8879669; 9634119-9639583; 9798899-9810311; 9580950-9588272; 9895078-9909248; 10610941-10615959; 12672652-12875736; 12773925-12774030; 12774750-12775838; 12776295-12776588; 12776699-12776933	8745688-8746162	D9S0242i
ZPLD1	131368	chr3	102153859-102198685	101693151-101727579; 101705690-101705940; 101706681-101707220; 101707358-101707582; 101735783-101736387; 101743662-101746954; 101793313-101803629; 101807405-101812716; 101937227-101965327; 101942425-101942749; 101943175-101943278; 101967606-101982707; 102014505-102021401; 102238084-102258547	102991462-102991819; 103914598-103915045	D3S0313i; D3S0923i

#: Refseq or GENCODE coordinate for the gene. When existing multiple transcripts, the longest is shown.

* 47 microsatellite markers are reported by Michikawa et al to be associated with radiosensitivity from a GWAS study (PMID: 20701746).

Table S2. 52 *SEMA3A* dependently differentially expressed genes in t-AML.

symbol	description	chr	band	CC (tAML & N)	FC (tAML>N)	CC (-7/del7 tAML & N)	FC (-7/del7 tAML>N)
CDA	cytidine deaminase	1	p36.12	0.6	3.5	0.7	3.4
CD1E	CD1e molecule	1	q23.1	0.6	3.2	0.7	3.0
CCDC181	coiled-coil domain containing 181	1	q24.2	0.6	2.6	0.7	3.3
PAPPA2	pappalysin 2	1	q25.2	0.7	3.3	0.7	3.5
CAMSAP2	calmodulin regulated spectrin-associated protein family, member 2	1	q32.1	0.8	3.2	0.8	4.0
EPHX1	epoxide hydrolase 1, microsomal (xenobiotic)	1	q42.12	0.7	2.7	0.8	3.0
EIF2AK2	eukaryotic translation initiation factor 2-alpha kinase 2	2	p22.2	0.7	4.0	0.7	3.7
DTNB	dystrobrevin, beta	2	p23.3	0.6	3.0	0.7	3.4
LCT	lactase	2	q21.3	0.7	3.5	0.5	2.7
GULP1	GULP, engulfment adaptor PTB domain containing 1	2	q32.1	0.8	5.3	0.7	4.4
MSTN	myostatin	2	q32.2	0.7	3.0	0.8	2.8
MYL1	myosin, light chain 1, alkali; skeletal, fast	2	q34	0.6	2.6	0.7	2.8
SORBS2	sorbin and SH3 domain containing 2	4	q35.1	0.7	3.3	0.7	3.1
HIST1H2BD	histone cluster 1, H2bd	6	p22.2	0.7	7.2	0.5	5.0
CAP2	CAP, adenylate cyclase-associated protein, 2 (yeast)	6	p22.3	0.7	4.2	0.6	4.5
ME1	malic enzyme 1, NADP(+)-dependent, cytosolic	6	q14.2	0.6	5.9	0.5	4.5
SIM1	single-minded family bHLH transcription factor 1	6	q16.3	0.7	3.0	0.6	2.9
GJA1	gap junction protein, alpha 1, 43kDa	6	q22.31	0.6	7.0	0.8	8.8
HOXA11	homeobox A11	7	p15.2	0.7	3.8	0.7	2.9
SEMA3A	sema domain, immunoglobulin domain (Ig), short basic domain, secreted, (semaphorin) 3A	7	q21.11	1.0	2.3	1.0	2.2
SLC18A1	solute carrier family 18 (vesicular monoamine transporter), member 1	8	p21.3	0.7	2.8	0.8	3.1
GDF10	growth differentiation factor 10	10	q11.22	0.6	2.9	0.6	2.9
BMPR1A	bone morphogenetic protein receptor, type IA	10	q23.2	0.7	2.9	0.8	4.2
CYP2C9	cytochrome P450, family 2, subfamily C, polypeptide 9	10	q23.33	0.7	4.3	0.7	4.4
P2RX3	purinergic receptor P2X, ligand-gated ion channel, 3	11	q12.1	0.6	3.3	0.6	3.4
ROM1	retinal outer segment membrane protein 1	11	q12.3	0.8	2.6	0.9	2.8
NRXN2	neurexin 2	11	q13.1	0.7	4.3	0.7	3.9
KCNJ1	potassium inwardly-rectifying channel, subfamily J, member 1	11	q24.3	0.7	3.0	0.8	3.3
PTHLH	parathyroid hormone-like hormone	12	p11.22	0.7	2.5	0.6	3.0
GYS2	glycogen synthase 2 (liver)	12	p12.1	0.7	4.8	0.7	5.0
GUCY2C	guanylate cyclase 2C (heat stable enterotoxin receptor)	12	p12.3	0.7	3.0	0.5	3.1
KLRC3	killer cell lectin-like receptor subfamily C, member 3	12	p13.2	0.8	2.9	0.8	2.9
KRT85	keratin 85	12	q13.13	0.7	3.4	0.7	3.7
KL	klotho	13	q13.1	0.7	2.8	0.5	2.8
MAB21L1	mab-21-like 1 (C. elegans)	13	q13.3	0.7	3.7	0.7	4.0
FEM1B	fem-1 homolog b (C. elegans)	15	q23	0.7	3.4	0.8	3.7
FURIN	furin (paired basic amino acid cleaving enzyme)	15	q26.1	0.6	3.2	0.5	3.2
TNFRSF17	tumor necrosis factor receptor superfamily, member 17	16	p13.13	0.7	2.6	0.6	2.7
ZNF174	zinc finger protein 174	16	p13.3	0.6	4.0	0.6	2.9
ADORA2B	adenosine A2b receptor	17	p12	0.7	3.1	0.7	3.2
VTN	vitronectin	17	q11.2	0.7	2.7	0.6	2.8
SEBOX	SEBOX Homeobox	17	q11.2	0.7	2.7	0.6	2.8
KRT34	keratin 34	17	q21.2	0.6	2.8	0.8	2.9
ABCA6	ATP-binding cassette, sub-family A (ABC1), member 6	17	q24.3	0.7	3.6	0.6	2.9
SERPINB7	serpin peptidase inhibitor, clade B (ovalbumin), member 7	18	q21.33	0.7	2.5	0.7	2.8
DNASE2	deoxyribonuclease II, lysosomal	19	p13.2	0.7	3.3	0.7	3.5
BBC3	BCL2 binding component 3	19	q13.32	0.8	3.0	0.7	3.2
KLK10	kallikrein-related peptidase 10	19	q13.41	0.6	3.8	0.6	3.1
SNPH	syntaphilin	20	p13	0.7	2.9	0.7	3.1
KIF3B	kinesin family member 3B	20	q11.21	0.7	3.7	0.7	4.4
BPI	bactericidal/permeability-increasing protein	20	q11.23	0.6	3.4	0.5	2.8
TMPRSS15	transmembrane protease, serine 15	21	q21.1	0.7	2.9	0.6	2.6
RAP2C	RAP2C, member of RAS oncogene family	X	q26.2	0.6	4.7	0.7	4.2

Legend: CC: correlation coefficient; FC: fold change; N: normal control.

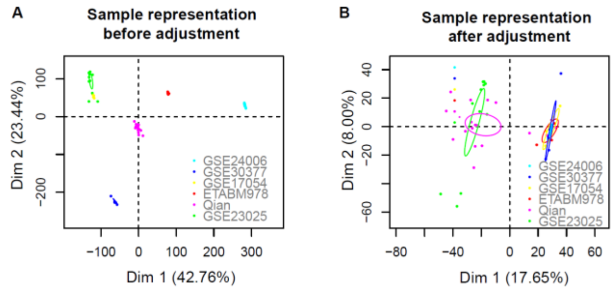

Figure S1. Correction of batch effects. A) There are batch effects when integrating samples from different datasets, showing by the first two principal components derived from all genes. B) The dataset-dependent batch effects are removed after the correction. In both panels, one dot is one sample colored by the datasets.

.

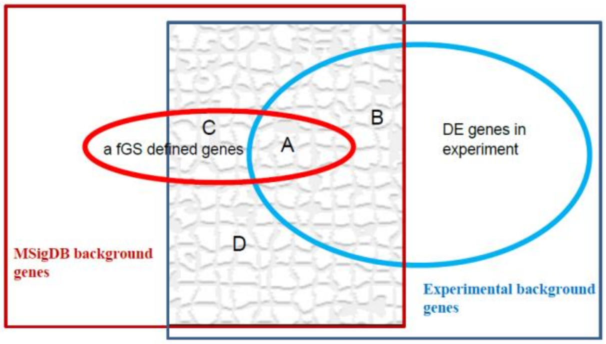

contingency table

	In fGS	Out fGS
DE	A	B
Not DE	C	D

$$p = \frac{(A+B)!(C+D)!(A+C)!(B+D)!}{A!B!C!D!\ (A+B+C+D)!}$$

Figure S2. Conditional hypergeometric distribution test. Note that the test uses the common genes (A+B+C+D, in gray) covered by both MSigDB and an experiment of interest. fGS: functional gene-set; DE: differentially expressed.
